# Rapid and Efficient Separation of Decursin and Decursinol Angelate from *Angelica gigas* Nakai using Ionic Liquid, (BMIm)BF_4_, Combined with Crystallization

**DOI:** 10.3390/molecules24132390

**Published:** 2019-06-28

**Authors:** Alice Nguvoko Kiyonga, Ji-Hun An, Ki Yong Lee, Changjin Lim, Young-Ger Suh, Young-Won Chin, Kiwon Jung

**Affiliations:** 1 College of Pharmacy, CHA University, Sungnam 13844, Korea; 2 College of Pharmacy, Korea University, Sejong City 30019, Korea; 3 College of Pharmacy, Dongguk University Seoul, Goyang-si, Gyeonggi-do 10326, Korea

**Keywords:** ionic liquids, decursin, decursinol angelate, *Angelica gigas* Nakai, crystallization

## Abstract

Ionic liquids (ILs) have gained much attention as alternative solvents to volatile organic solvents due to their attractive properties. This study aimed to develop an efficient method for the selective separation of decursin (D) and decursinol angelate (DA) from *Angelica gigas* Nakai (*A. gigas*) using ILs and crystallization. The IL 1-butyl-3-methylimidazolium tetrafluoroborate ((BMIm)BF_4_) was the most efficient at extracting D and DA. Parameters including solid-to-liquid ratio, time, and temperature were optimized by response surface methodology (RSM). Under optimal extraction conditions (1 g/6.5 mL solid-to-liquid ratio, 60 °C temperature, and 120 min time), the extraction yields of D and DA were 43.32 mg/g (97.06%) and 17.87 mg/g (97.12%), respectively. Moreover, drowning out crystallization using deionized water (DW) as an anti-solvent offered an excellent ability to recover D and DA from the *A. gigas*–(BMIm)BF_4_ extraction solution. The rates of recovery and the total purity of D and DA were found to be greater than 97%. Therefore, a rapid and efficient method of combining ILs with crystallization was effectively achieved for the selective separation of D and DA. This approach is assumed to be beneficial in the pharmaceutical industry for the effective obtention of D- and DA-enriched products.

## 1. Introduction

**Figure 1:**
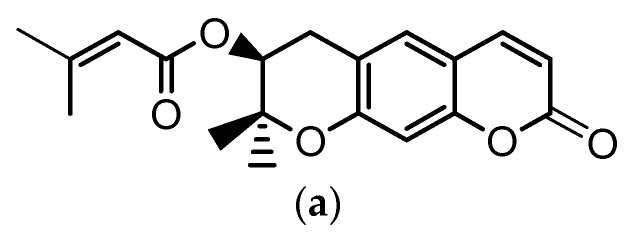

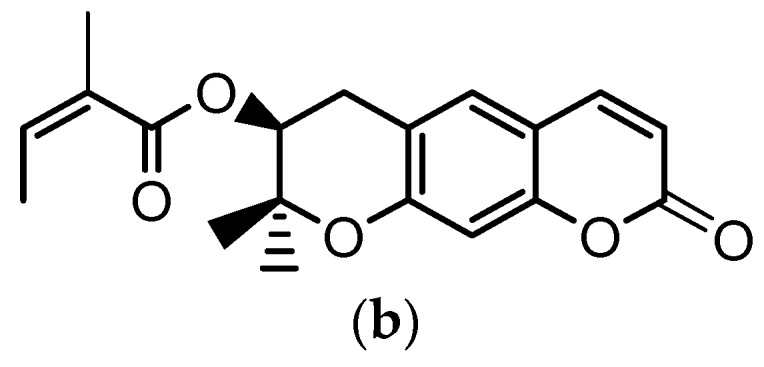
Chemical structures of: (**a**) decursin (D) and (**b**) decursinol angelate (DA).

*Angelica gigas* Nakai (*A. gigas)* is a Korean medicinal herb for which the dried roots have been extensively used in the Korean pharmacopoeia for the treatment of several diseases such as neuralgia, headache, cold, arthralgia, gynecological disorders, etc. The pyranocumarins, decursin (D) (Figure 1a) and decursinol angelate (DA) (Figure 1b) are reported as the principal bioactive compounds of this plant [1,2,3].

Recent studies have reported that D and DA exhibit significant effects on colon cancer [4], breast cancer [5], Alzheimer’s disease [6], etc. [7,8,9]. Hence, studies have been intensively carried out to investigate the potential effects of D and DA on various diseases. Moreover, various techniques for the separation of D and DA, including microwave-assisted extraction [10], accelerated solvent extraction [11], etc. [12,13,14,15] have been reported as well. However, most of methods developed for the separation of D and DA are time consuming, tedious, labor-intensive, and involve repetitive steps, and thus, require a high consumption of volatile organic solvents. Therefore, in recent decades, studies have been conducted to remedy the shortcomings of conventional method employed for D and DA separation. Nevertheless, studies involving the development of an industrially suitable and efficient method for the selective separation of D and DA have yet not been reported. Therefore, the development of a novel approach able to improve the extraction yield, recovery, as well as the purity of D and DA is urgently needed.

In the past few decades, ionic liquids (ILs) have received considerable attention as alternative solvents to volatile organic solvents for the extraction and isolation of natural bioactive compounds. Ionic liquids is a term that refers to salts with a melting point below 100 °C and which are entirely composed of ions [16,17]. Ionic liquids possess unique and attractive properties including high selectivity, negligible vapor pressure, low volatility and inflammability, large liquid range, great chemical and thermal stability, strong solubility power and possible tunability by varying the IL’s cation and anion [18,19]. In regards to their unique properties, various studies involving the applicability of ILs as solvents for the separation of phenolic compounds [20], alkaloids [21], ginsenosides [22], and other phytochemicals [23,24,25,26,27,28] have been conducted. However, due to their exceptional synthesis process, their expensive unit price, and their innumerable kinds, over 10^18^, studies related to the applicability of ILs in the isolation of natural products are yet insignificant.

Besides, separation techniques using supercritical carbon dioxide [29], pervaporation [30], microporous resins [31], liquid–liquid extraction [32,33,34], as well as crystallization [35,36] methods have been successfully applied in combination with ILs for the recovery of target compounds from the IL extraction solutions. Crystallization is an excellent separation technique because it can efficiently and easily produce single purified compounds. It is also crucial to improve the quality of obtained products [37,38,39,40]. In 2006, Lapkin et al. investigated the isolation of artemisinin, a malaria drug, using ILs as solvent and utilizing crystallization techniques. Herein, artemisinin was obtained with a high purity of 95% and the yield increased up to 82% [36].

Therefore, the present study was carried out to develop a suitable and effective method for the selective separation of D and DA from *A. gigas* using ILs as extraction solvent. The objective of this study was to confirm the potentiality and importance of ILs as new alternative solvents for the separation of natural compounds as well. The extraction time, temperature, and solid-to-liquid ratio were the parameters taken into consideration. Response surface methodology (RSM) with a Box–Behnken design was applied to investigate the interaction between the above parameters and to determine their influence on the extraction yields of D and DA. Further, drowning out crystallization was applied to recover D and DA from the *A. gigas*–IL extraction solutions using different anti-solvents. To our knowledge this was the first time a method combining ILs and crystallization has been systematically investigated and successfully achieved for the separation of D and DA from *A. gigas*.

## 2. Results and Discussion

### 2.1. Screening of ILs

**Figure 2:**
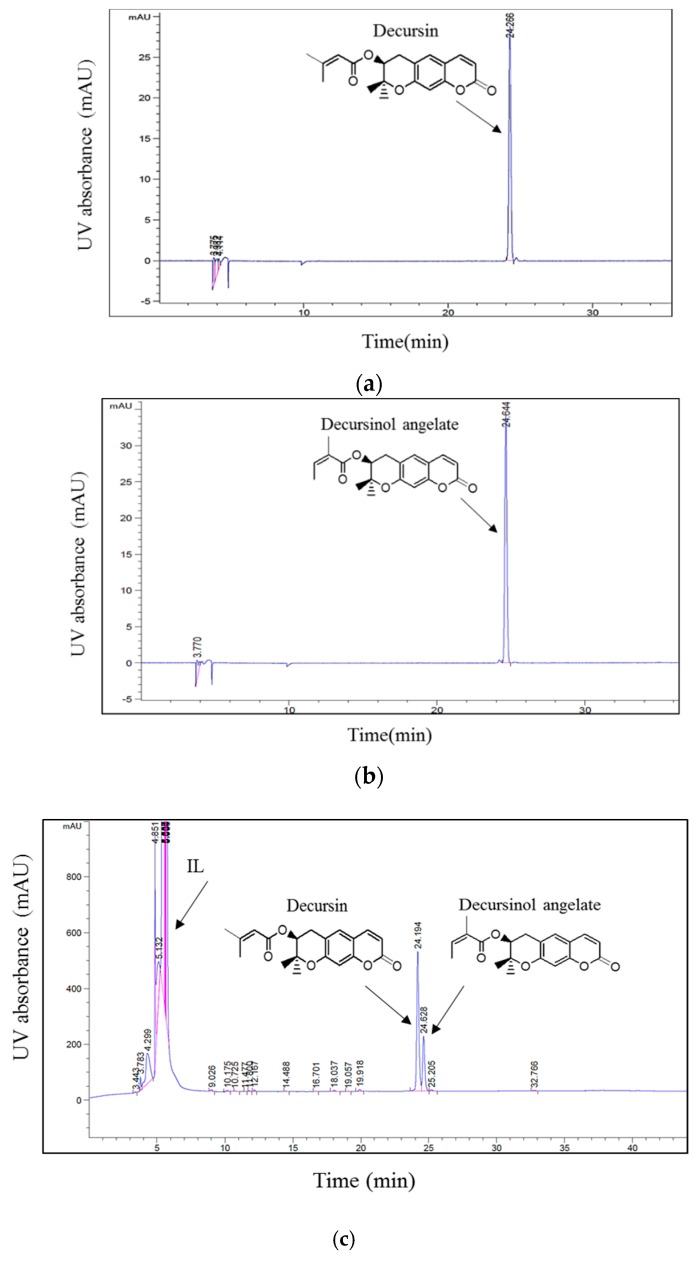
HPLC chromatograms of (**a**) D standard, (**b**) DA standard, (**c**) *A. gigas*–ionic liquid (IL) extraction solution. (UV 210 nm).

Because of their unique and attractive properties, ILs have been efficiently applied in the separation of several bioactive compounds from plant materials. Moreover, previous studies have suggested that parameters including the structures of ILs, the cations, and/or anions of ILs influence the extraction efficiency of specific compounds [25,26]. In their study, Han et al. [20], suggested that the cation and anion have a large influence on the extraction efficiency of phenolic compounds from *Laminaria japonica* Aresch. In this regard, three ILs containing 1-butyl-3-methylimidazolium as cation and Tf_2_N^−^, PF_6_^−^, and BF_4_^−^ as anions were evaluated to examine the effect of these anions on the simultaneous extraction of D and DA. The extraction experiment was performed by stirring 1 g of dried and powdered roots of *A. gigas* with a known volume (6 mL) of ILs at room temperature (20 °C) and 500 rpm for 2 h. Afterwards, the extracts were filtered and then analyzed via HPLC according to the HPLC method depicted in Section 3.2. The chromatographic peaks of D and DA in the *A. gigas*–IL extraction solutions were confirmed by comparing their retention times and UV spectra with those of the reference standards in Figure 2.

**Figure 3:**
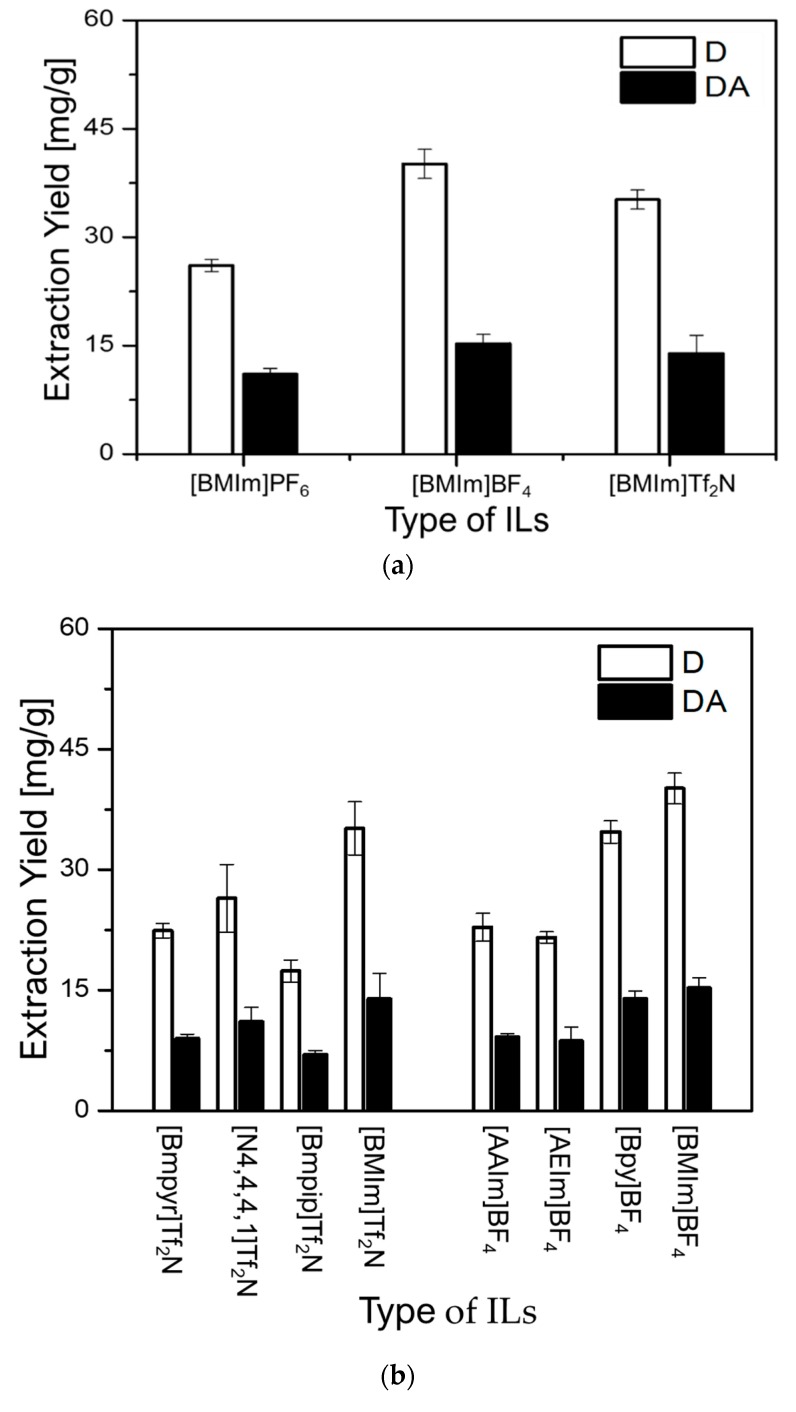
Effect of ILs on the extraction yields of D and DA: (**a**) effect of anions and (**b**) effect of cations.

As can be observed in Figure 3a, (BMIm)BF_4_ revealed the highest extraction capacity for both D (D = 40.12 mg/g) and DA (DA = 15.27 mg/g), followed by (BMIm)Tf_2_N (D = 35.00 mg/g and DA = 13.90 mg/g) and (BMIm)PF_6_ (D = 26.10 mg/g and DA = 11.06 mg/g), respectively. Because, (BMIm)BF_4_ and (BMIm)Tf_2_N demonstrated better extraction efficiencies for both D and DA, their respective anions BF_4_^−^ and Tf_2_N^−^ were further investigated. Using these anions, BF_4_^–^ and Tf_2_N^−^, ILs with distinct cations were evaluated to investigate the effect of their cations on the extraction yields of D and DA. As can be seen in Figure 3b, the greatest extraction ability of 40.12 mg/g for D and 15.27 mg/g for DA was obtained with (BMIm)BF_4_. It was also found that (Bpy)BF_4_ (D = 34.70 mg/g and DA = 13.94 mg/g), and (BMIm)Tf_2_N (D = 35.00 mg/g and DA = 13.90 mg/g) possess similar ability to extract both D and DA. However, other ILs showed very low ability to extract D and DA. Based on these results, it was concluded that the cations and anions of ILs simultaneously influence the extraction yields of D and DA.

### 2.2. Single Factor Experiments

#### 2.2.1. Effect of Extraction Time

**Figure 4:**
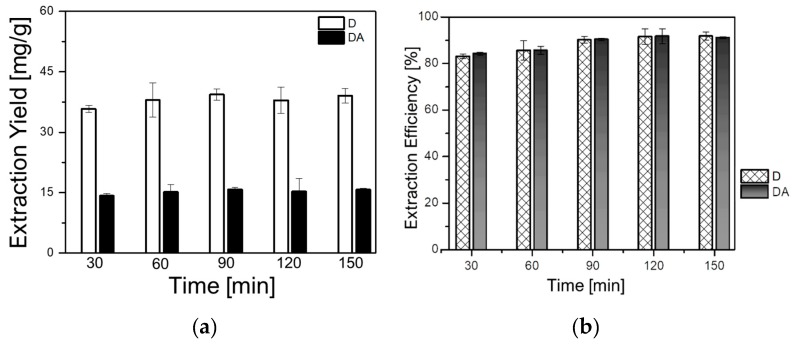
Effect of the extraction time on: (**a**) extraction yields of D and DA and (**b**) extraction efficiencies of D and DA.

The impact of time on the extraction yields of D and DA was investigated to optimize the extraction time, and thus, to avoid time consuming and incomplete extraction. Five samples of 1 g each were extracted with (BMIm)BF_4_ at 30 °C, the solid-to-liquid ratio was fixed to 1 g/6 mL and the rotational speed was 500 rpm. The time parameter was varied as follows: 30, 60, 90, 120, and 150 min. As can be seen in Figure 4a,b, the yield of D increased from 38.26 mg/g (85.72%) at 30 min to 40.99 mg/g (90.02%) at 90 min. In addition, the yield of DA increased from 15.68 mg/g (85.22%) at 30 min to 17.07 mg/g (92.79%) at 90 min. No significant increment in the yield of either D or DA could be observed with the increase of the extraction time. Thus, 90 min was selected as appropriate extraction time for further experiments.

#### 2.2.2. Effect of Solid-to-Liquid Ratio

**Figure 5:**
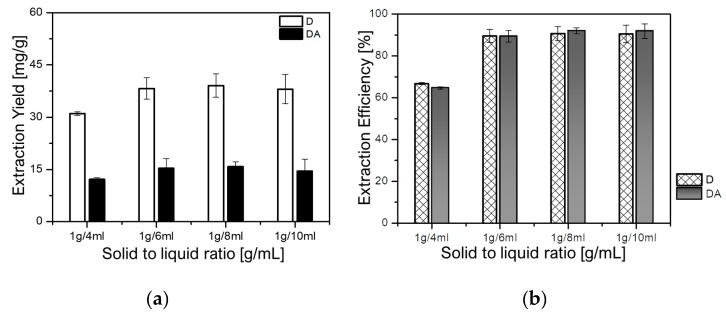
Effect of the solid-to-liquid ratio on: (**a**) extraction yields of D and DA and (**b**) extraction efficiencies of D and DA.

The effect of the solid-to-liquid ratio was evaluated to determine the optimum ratio for maximizing the extraction yield of D and DA, thus, avoiding both solvent waste and incomplete extraction of target components [25]. In this study, four samples of 1 g each were individually extracted at fixed temperature of 30 °C, and rotational speed of 500 rpm for 90 min. The solid-to-liquid ratio was set in the range from 1 g/4 mL to 1g/10 mL to evaluate the effect of the solid-to-liquid ratio on the extraction yields of D and DA. As depicted in Figure 5a,b, the yields of D dramatically increased from 29.80 mg/g (66.77%) at the ratios of 1 g/4 mL to 40.49 mg/g (90.73%) at 1 g/8 mL. While that of DA dramatically increased from 11.90 mg/g (64.68%) to 16.93 mg/g (92.01%) at the ratios of 1g/4 mL and 1 g/8 mL, respectively. However, a slight decrease in the yields of both D and DA, respectively, 40.40 mg/g (90.53%) and 15.53 mg/g (84.42%), could be observed when the solid-to-liquid ratio was 1 g/10 mL. Hence, 1 g/8 mL was selected as an appropriate solid-to-liquid ratio for further analysis.

#### 2.2.3. Effect of Temperature

**Figure 6:**
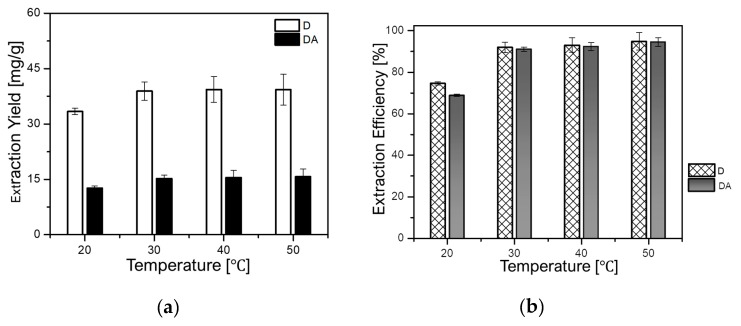
Effect of temperature on: (**a**) extraction yields of D and DA and (**b**) extraction efficiencies of D and DA.

It is well known that the rise in temperature can influence the extraction yield of target compounds by enhancing their solubility into the solvent. However, the increment of temperature can also cause degradation of heat-sensible compounds. Therefore, it is of great importance to investigate the impact of temperature on the extraction of target compounds. In this regard, the influence of temperature on the extraction yields of D and DA was evaluated at a constant rotational speed of 500 rpm and a fixed solid-to-liquid ratio of 1 g/8 mL for 90 min. The temperature was varied as follows: 20, 30, 40, and 50 °C. As depicted in Figure 6a,b, the yield of D augmented from 33.30 mg/g (74.62%) to 42.33 mg/g (94.84%), while that of DA increased from 12.67 mg/g (68.85%) to 17.26 mg/g (93.80%) when the temperature was set from 20 °C to 50 °C, respectively.

### 2.3. Optimization of Extraction Condition by Response Surface Method (RSM)

Fifteen runs were conducted using RSM (Box–Behnken experimental design) to investigate the interaction between three variables: extraction time, temperature, and the solid-to-liquid ratio. The extraction time ranged from 30 to 120 min, the temperature from 20 to 60 °C, and the solid-to-liquid ratio from 1 g/4 mL to 1 g/8 mL. These conditions were set based on the results obtained from the previous section. The experimental conditions as well as the obtained responses are depicted in Table 1. In addition, below are the second-order polynomial equations for the determination of the extraction yields of D (Y_D_) and DA (Y_DA_); Y_D_ = −81.4555 + 0.309886A + 0.686104B + 79.2225C + 2.2428A^2^ − 0.011615B^2^ − 11.2171C^2^ + 0.00096AB − 0.056AC + 0.08575BC (Actual *R*^2^ = 99.33% and Adjusted *R*^2^ = 98.12%) and Y_DA_ = −94.5399 + 0.4458A + 0.812312B + 83.5383C − 1.79012A^2^ − 0.0109156B^2^ − 11.6538C^2^ − 3.7500AB − 0.07844AC + 0.0822500BC (Actual *R*^2^ = 99.27% and Adjusted *R*^2^ = 97.96%). Herein, A represents the extraction time, B the temperature, and C the solid-to-liquid ratio. The regression coefficient (*R*^2^) and the probability (*p*-value) were used to confirm the quality and significance of the results.

Table 2 List of the *F*-values and *p*-values calculated from the model. As can be seen in Table 2, the *F*-values for D and DA were 82.02 and 75.65, respectively, and the *p*-values were inferior to 0.0001. The observed high *F*-values and the lower *p*-values indicate that the developed model was significant and that the chance for the model *F*-value of this size to occur as the statistical noise was less than 0.0001%. The values of “lack of fit” of *p*-values and *F*-values (0.117 and 7.72 for D, and 0.117 and 11.85 for DA, respectively) indicate that the lack of fit was not significant. Generally, values of probability less than 0.05 or greater than 0.01 indicate that the model terms are significant or not significant. In this model, linear coefficients A, B, and C; quadratic term coefficients B^2^ and C^2^, and cross product coefficients AC and BC were significant with *p*-values inferior to 0.05. However, quadratic term coefficient A^2^ and cross product coefficient AB were not significant. Based on the overall obtained results, the model was confirmed very satisfactory.

**Figure 7:**
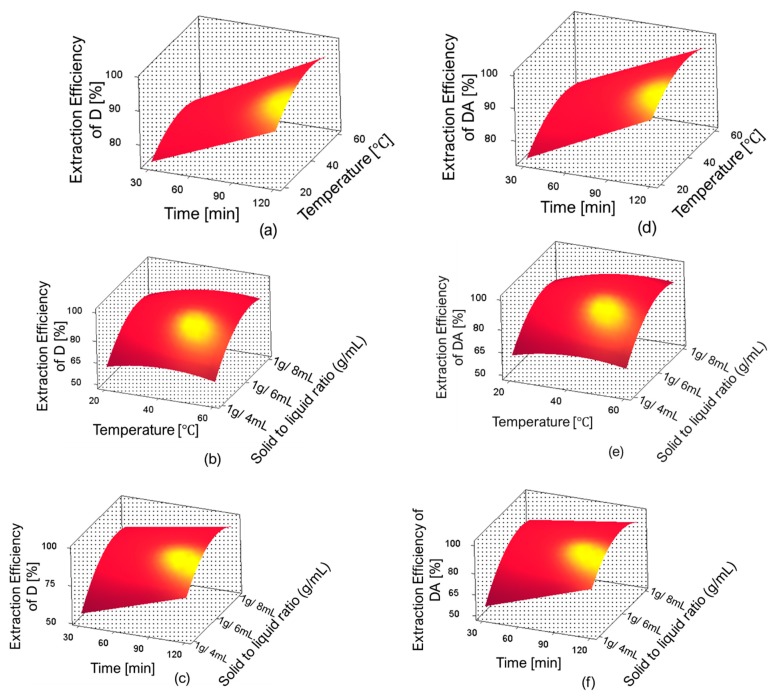
Response surface plots showing the interaction between single factors on the extraction efficiencies of D and DA. (**a**) effect of time and temperature on extraction efficiency of D, (**b**) effect of temperature and solid-to-liquid ratio on extraction efficiency of D, (**c**) effect of time and solid-to-liquid ratio on extraction efficiency of D, (**d**) effect of time and temperature on extraction efficiency of DA, (**e**) effect of temperature and solid-to-liquid ratio on extraction efficiency of DA and (**f**) effect of time and solid-to-liquid ratio on extraction efficiency of DA.

The three-dimensional response surface plots for D and DA extraction efficiency are shown in Figure 7. In these figures, one can observe the influence of independent variables and their interactions on the extraction efficiencies of D and DA. The optimum extraction conditions proposed by the RSM were: extraction time 120 min, temperature 59.98 °C, and solid-to-liquid ratio 1 g/6.46 mL; the predicted extraction yields were 43.74 mg/g (98%) for D and 18.03 mg/g (97.99%) for DA. However, the predicted conditions were slightly adjusted for the validation experiments as follows: extraction time 120 min, temperature 60 °C, and solid-to-liquid ratio 1 g/6.5 mL.

### 2.4. Verification of the Optimal Extraction Method

In order to evaluate the reproducibility and precision of the new developed extraction method, three samples of 1 g each were extracted under the adjusted optimal condition (extraction time 120 min, temperature 60 °C, and solid-to-liquid ratio 1 g/6.5 mL) predicted by RSM. The results (Table 3) show that the yields of D and DA were 43.32 mg/g (97.09%) and 17.87 mg/g (97.07%), respectively. Based on these results, it can be assessed that the obtained experimental results are in great accordance with the results predicted by the RSM model (respectively, 43.74 mg/g for D and 18.03 mg/g for DA). Moreover, the experiments showed a good reproducibility and precision with RSD of 0.13% and 0.14% for the D and DA, respectively.

### 2.5. Comparison of the (BMIm)BF_4_ Extraction Method with other Extraction Methods

The (BMIm)BF_4_ extraction method was compared with other methods employed for the extraction of D and DA. Herein, *A. gigas* was extracted according to Lee’s (2006) conventional reflux method (reflux extraction condition: 60% EtOH, temperature 95 °C, extraction time 6 h) [9] and Kang’s (2003) extraction method (extraction condition: 50% EtOH, temperature 60 °C, time 5 h) [12]. Besides, *A. gigas*. was extracted with 100% EtOH at the same extraction conditions with that of (BMIm)BF_4_ (extraction time 120 min, temperature 60 °C, and solid-to-liquid ratio 1 g/6.5 mL). The results illustrated in Table 4 reveal that the extraction yields of D and DA were greater with the (BMIm)BF_4_ IL extraction method compared to other methods. The extraction time was also shorter than in other methods. Thus, it was assessed that the IL-based extraction method was most efficient in terms of the extraction yields of D and DA, and required less extraction time and solvent consumption when compared to other methods.

### 2.6. Drowning out Crystallization of D and DA from A. gigas–(BMIm)BF_4_ Extraction Solution

Drowning out crystallization experiments were performed in order to recover D and DA from the *A. gigas*–(BMIm)BF_4_ extraction solutions. Herein, the experiments were conducted using various anti-solvents (MeOH, EtOH, EA, IPA, and DW) at a randomly selected *A. gigas*–IL extraction solution to anti-solvents ratio of 1:5 (*v*/*v*) at 20 °C and 200 rpm for 5 h. As result, crystal could not be yielded when MeOH, EtOH, EA, and IPA were used as anti-solvents. However, precipitates were obtained when DW was used as an anti-solvent. The obtained precipitates were analyzed by means of HPLC to determine whether D and DA were recovered well from the IL extraction solutions and to evaluate their purities. From the HPLC analysis results, the rates of recovery of D and DA as well as their total purity exceeded 80%.

Furthermore, the correlation between the supersaturation level and the selective crystallization of D and DA was investigated to determine the reason influencing the occurrence and non-occurrence of D and DA crystallization when different anti-solvents were utilized. Supersaturation is the difference between the initial and the equilibrium concentration (solubility) of a certain compound. It is assumed as the driving force to induce crystallization. The supersaturation can be expressed as supersaturation level (S) and expressed as S~C_I_/C_eq_. Here, C_I_ indicates the initial concentration and C_eq_ the equilibrium concentration. Generally, spontaneous crystallization occurs at supersaturated state when S > 1; however, when S < 1, it is an undersaturated state; therefore, a spontaneous crystallization cannot be induced. Additionally, crystallization is impossible when S = 1 because the solution is in the saturated state, at this state, equilibrium is maintained. This shows that crystallization can occur only at a supersaturation level consistently higher than 1 [38,39]. Table 5 presents different values of D and DA supersaturation levels (S_D_ and S_DA_) when different anti-solvents (MeOH, EtOH, and DW) were used. The result revealed the reason why crystallization was possible exclusively when DW was applied as anti-solvent. The fact that S_D_ and S_DA_ were lower when MeOH and EtOH were used as anti-solvents is presumed as the causes leading to the non-occurrence of precipitation. However, the high supersaturation levels, S_D_ = 5.28 and S_DA_ = 46.6, respectively, observed for D and DA when DW was the anti-solvent, impacted the crystallization of D and DA from the IL extraction solutions. This is because these supersaturation levels were satisfactory to induce spontaneous nucleation of target compounds, and consequently, their crystallization. Moreover, the simultaneous high supersaturation levels observed for both D and DA were assumed to influence their simultaneous nucleation, thus, the obtention of D and DA solid mixture.

**Figure 8:**
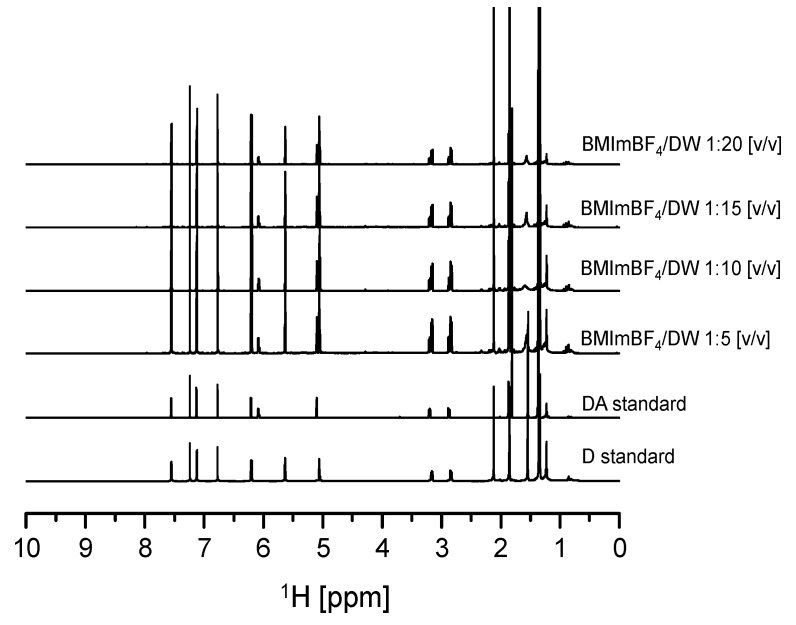
Solution-state ^1^H-NMR spectra for D and DA precipitated from *A. gigas*–(BMIm) BF_4_ extraction solution at different DW ratios (*v*/*v*) (CDCl3).

In addition, drowning out crystallization experiments were carried out using various ratios of *A. gigas*–(BMIm)BF4 extraction solution to DW compositions (1:1, 1:3, 1:5, 1:10, 1:15, 1:20) to evaluate the effect of DW composition ratios on the crystallization behavior and recovery of both D and DA. The initial concentrations of D and DA in the *A. gigas*–(BMIm)BF4 extraction solution were 6.22 mg/mL and 2.631 mg/mL, respectively. The temperature and the rotational speed were 20 °C and 200 rpm, respectively. From the results, it was found that when the ratio of *A. gigas*–(BMIm)BF_4_ extraction solution to DW was set to 1:1 (*v*/*v*), precipitates could not be induced even after three days of solution stirring. In addition, when the ratio of *A. gigas*–(BMIm)BF_4_ extraction solution to DW was set to 1:3 (*v*/*v*), precipitates were obtained after 20 h of solution stirring: however, in a much lower yield. Nevertheless, when the ratios of *A. gigas*–(BMIm)BF_4_ extraction solution to DW were set to 1:5, 1:10, 1:15, and 1:20 (*v*/*v*), large quantities of solid materials could be induced very rapidly within only one to five hours of stirring. The obtained solid materials were analyzed via solution-state 1H-NMR as presented in Figure 8. From the solution-state ^1^H-NMR data presented in Figure 8, it was ascertained that the solid materials obtained in this work were mixtures of sole D and DA precipitates.

The Table 6 presents the supersaturation level profile for D and DA monitored when the crystallization was induced at different ratios (1:1, 1:3, 1:5, 1:10, 1:15, and 1:20) of *A. gigas*–(BMIm)BF_4_ extraction solution to DW. These results deduced the reason leading to the non-occurrence of crystallization at the ratio 1:1 and to the occurrence of small product quantity at the ratio 1:3. As seen in Table 6, at the ratio of 1:1, the S_D_ and S_DA_ were 1.01 and 1.07, respectively. Although, these supersaturation levels (S) were greater than 1 (S > 1), they are assumed inadequate to induce spontaneous nucleation of D and DA, and consequently, the reason for the non-occurrence of crystallization at this ratio. In addition, the supersaturation levels (S) for D and DA were S_D_ = 1.77 and S_DA_ = 1.83, respectively, at 1:3 ratio. These supersaturation levels (S) were considered capable to induce spontaneous nucleation of both D and DA. Thus, crystals formation was possible at this ratio. Moreover, as shown in Table 6, very high supersaturation levels (S), exceeding 4.5, were monitored for both D and DA at 1:5, 1:10, 1:15, and 1:20 ratios. Hence, the large supersaturation levels observed at these composition ratios were assumed to provoke the rapid and simultaneous precipitation of D and DA.

Table 7 illustrates the rates of recovery of D and DA at different ratios (1:3, 1:5, 1:10, 1:15, and 1:20) of *A. gigas*–(BMIm)BF_4_ extraction solution to DW. The obtained result showed that crystallization was not induced when the ratio was 1:3; however, it was greatly achieved at ratios greater than 1:5. As can be seen in Table 7, the rates of recovery of D dramatically improved from 43.41% to 79.74%, and those of DA dramatically improved from 45.25% to 81.75% at 1:3 and 1:5 ratios, respectively. The rates of recovery of D increased from 90.35% to 97.42% when the ratios were set to 1:10 and 1:15, respectively. Also, the rates of recovery of DA increased from 95.82% to 97.71% at ratios of 1:10 and 1:15, respectively. At last, the rates of recovery of D and DA at 1:20 were recorded as D = 96.62% and DA = 98.09%, respectively. The increase of D and DA recovery rates was attributed to the change in their supersaturation levels when the ratio of DW was increased (Table 6).

Moreover, the total purity of D and DA in products obtained at 1:5, 1:10, 1:15, and 1:20 ratios of *A. gigas*-(BMIm)BF_4_ extraction solution to DW was determined. These ratios were selected as they demonstrated high recovery of D and DA. The results are illustrated in Table 8. As can be observed in Table 8, the average total purity of D and DA was of approximatively 97% in the products.

**Scheme 1:**
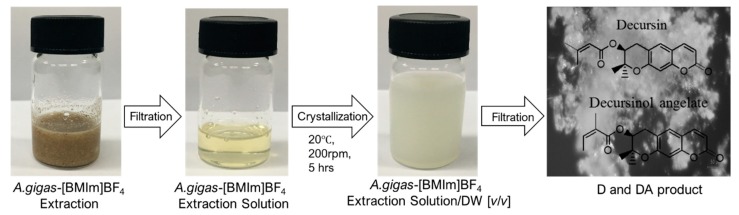
Scheme for separating D and DA from *A. gigas* using (BMIm)BF_4_ and crystallization.

From this study, an efficient and adequate method for the rapid and selective separation of D and DA from *A. gigas* using (BMIm)BF_4_ as extraction solvent and DW as anti-solvent was developed. Only these ratios of *A. gigas*–(BMIm)BF_4_ extraction solution to DW, 1:5 1:10, 1:15, and 1:20, were found favorable to produce D and DA with high yields and high purities. The separation scheme of D and DA from *A. gigas* using IL ((BMIm)BF_4_) as extraction solvent and drowning out crystallization for the recovery of D and DA is illustrated in the Scheme 1.

## 3. Materials and Methods

### 3.1. Reagents and Materials

Decursin (D) and decursinol angelate (DA) standards were purchased from Chengdu Biopurify Phytochemicals Ltd. (China), and their purities were 98.12% and 98.27%, respectively. The roots of *Angelica gigas* Nakai (*A. gigas*) were purchased from a local market in Seoul (2017) and identified by Professor Jinwoong Kim of Seoul National University. A voucher (GEL-AG02) was deposited in the Herbarium of the College of Pharmacy, Seoul National University (Seoul, South Korea). Ionic liquids including 1-butyl-1-methylpyrrolidinium bis(trifluoromethanesulfonyl) imide (Bmpyr)Tf_2_N, 1-butyl-1-methylpiperidinium bis(trifluoromethanesulfonyl) imide (Bmpip)Tf_2_N, tributylmethylammonium bis(trifluoromethanesulfonyl) imide (N4,4,4,1)Tf_2_N, 1-allyl-3-ethylimidazolium tetrafluoroborate (AEIm)BF_4_, 1,3-diallylimidazolium tetrafluoroborate (AAIm)BF_4_, and 1-butylpyridinium tetrafluoroborate (Bpy)BF_4_ were purchased from Kanto Chem Co., Inc. (Tokyo, Japan). 1-butyl-3-methylimidazolium bis(trifluoromethylsulfonyl) imide (BMIm)Tf_2_N, 1-butyl-3-methylimidazolium hexafluorophosphate (BMIm) PF_6_ and 1-butyl-3-methylimidazolium tetrafluoroburate (BMIm)BF_4_ were purchased from A-star Co., Ltd. (Ulsan, Korea). The HPLC grade deionized water (DW), acetonitrile (ACN), phosphoric acid (purity > 99%), analytical grade methanol (MeOH), ethanol (EtOH) (purity > 98%), ethyl acetate (EA), and isopropyl alcohol (IPA) were all purchased from DaeJung Chem. Co., Ltd. (Siheung, South Korea).

### 3.2. Methods

#### 3.2.1. Extraction Procedure and Determination of Extraction Yields

The dried roots of *A. gigas* were uniformly grounded and sieved through a 50 mesh sieve (particle size < 300 μm). Nine samples of 1 g each were extracted with a known volume of different ILs such as (Bmpyr)Tf_2_N, (Bmpip)Tf_2_N, (N4,4,4,1)Tf_2_N, (BMIm)Tf_2_N, (BMIm)PF_6_, (BMIm)BF_4_, (AEIm)BF_4_, (AAIm)BF_4_, and (Bpy)BF_4_ at room temperature (20 °C) and constant rotational speed (500 rpm) for 2 h. The purpose was to evaluate the ability of distinct ILs (the effects of their anions and cations) on the extraction yields of D and DA, and to select the relevant IL. After extraction, all samples were subjected to vacuum filtration to collect the *A. gigas*–IL extraction solutions which were then analyzed via HPLC to determine the contents of D and DA in the extracts. After selecting the relevant IL, the abovementioned parameters were cautiously examined to determine the optimal extraction condition for maximizing the extraction yields of D and DA. Each experiment was conducted in triplicate. The extraction yield was calculated using the following equation:

(1) $$\mathrm{Extraction}\;\mathrm{yield}\;\left(\frac{\mathrm{mg}}{g}\right)=\frac{\mathrm{Aount}\;\mathrm{of}\;D\;\mathrm{or}\;\mathrm{DA}\;\mathrm{in}\;\mathrm{the}\;\mathrm{extract}\;\left(\mathrm{mg}\right)}{\mathrm{Total}\;\mathrm{amount}\;\mathrm{of}\;D\;\mathrm{or}\;\mathrm{DA}\;\mathrm{in}\;\mathrm{dry}\;\mathrm{weight}\;\mathrm{mass}\;\mathrm{of}\;A.gigas\;\left(g\right)}$$

#### 3.2.2. Optimization of the Extraction Method using Response Surface Methodology (RSM)

In order to maximize the extraction yields of D and DA, interaction between variables was investigated and optimized by response surface methodology employing Box–Behnken design of Minitab 16 software (Minitab LLC, PA, USA). The experimental factors were set based on the experimental results obtained from the single factors experiments, as follows: extraction time (30, 75, and 120 min), temperature (20, 40, and 60 °C) and solid-to-solvent ratio (1 g/4 mL, 1 g/6 mL, and 1 g/8 mL). The significance level was set at *p* < 0.05. The extraction yield was calculated using Equation (1).

#### 3.2.3. Sample Solutions Preparation

After extraction, all samples were subjected to vacuum filtration to collect the *A. gigas*–(BMIm)BF_4_ extraction solution. Afterwards, 10 μL of each filtrate was diluted with 100% of MeOH to obtain sample solutions of known concentration. The prepared solutions were filtered through 0.45 μm membrane filter and then injected into the HPLC system. The HPLC analysis experiments were performed in triplicate.

#### 3.2.4. High-Performance Liquid Chromatographic (HPLC) Measurements for the Quantitative Determination of D and DA

An Agilent HPLC equipment (Agilent 1260, Santa Clara, CA, USA) was used to determine the total extraction amounts and concentrations of D and DA. A Hydrosphere C-18 column (250 × 4.6 mm, 5 μm, 12 nm, YMC, Kyoto, Japan) was used in this analysis. The mobile phase composition was DW (A) and ACN (B) under the gradient elution condition of: 0.00–5.00 min, 15–30% B; 5.10–30.00 min, 50–75% B; 30.10–40.00 min, 75% B; 40.10–50.00 min, 15% B. The flow rate and the injection volume were 0.8 mL/min and 10 μL, respectively. The detection absorbance was 330 nm, while the column temperature and the sample running time were 30 °C and 50 min, respectively. The chromatographic peaks of D and DA contained in the *A. gigas*–IL extraction solutions were confirmed by comparing their retention times and UV spectra with those of the reference standards and was determined by the regression equations of the calibration curves. The calibration curves constructed on three consecutive runs exhibited a good linearity (*R*^2^ = 0.996, *n* = 7 for D and *R*^2^ = 0.999, *n* = 7 for DA) in the range of 2.5 to 100 μg/mL. The limit of detection (LOD) and the limit of quantification (LOQ) were, respectively, 0.316 μg and 0.958 μg for D and 0.764 μg and 2.316 μg for DA (Table 9).

#### 3.2.5. Quantitative Determination of D and DA in the Roots of *A. gigas*

In order to determine the total amounts of D and DA in the roots of *A. gigas* roots, two samples of *A. gigas* roots of 1 g each were extracted using 100 mL of 95% ethanol (EtOH) for 12 h at room temperature. The experiment was triplicated, and then, the collected solutions of EtOH were separately mixed and concentrated in vacuo for subsequent HPLC analysis. From this study, the obtained contents of D and DA in 1 g of *A. gigas* were, respectively, 44.63 mg/g (4.46% of *A. gigas* raw material) and 18.4 mg/g (1.84% of *A. gigas* raw material). Thus, these respective contents of D and DA (44.63 mg/g and 18.40 mg/g) were considered as reference (100% content) for further comparative experiments.

#### 3.2.6. Selective Crystallization of D and DA from A. *gigas*–IL Extraction Solution

Drowning out crystallization experiments were conducted to recover D and DA from the *A. gigas*–IL extraction solution. However, crystal formation as well as a rapid and effective recovery of D and DA could be observed exclusively from (BMIm)BF_4_ when DW was used as an anti-solvent. The obtained crystals were subjected to solution-state ^1^H-NMR measurements to confirm the nature of the obtained crystals. Afterwards, crystallization experiments were carried out at fixed *A. gigas*–IL extraction solution and varied DW anti-solvent fractions for 5 h at room temperature (20 °C) and 200 rpm for investigating the effect of DW ratios on the selective crystallization and recovery rate of D and DA. The ratio of the solvent to anti-solvent, *A. gigas*–IL extraction solution to DW, was set to 1:1, 1:3, 1:5, 1:10, 1:15, and 1:20 (*v*/*v*). All obtained crystals were thoroughly washed to remove the remaining IL, dried in vacuo at 40 °C for 24 h, and then stored at 4 °C prior to the analysis. The rate of recovery and purity of D and DA were determined by the following equations:

(2) $$\mathrm{Recovery}\;\left(\%\right)=\frac{\mathrm{Amount}\;\mathrm{of}\;D\;\mathrm{or}\;\mathrm{DA}\;\mathrm{in}\;\mathrm{the}\;\mathrm{product}\;\left(\mathrm{mg}\right)}{\mathrm{Initial}\;\mathrm{amount}\;\mathrm{of}\;D\;\mathrm{or}\;\mathrm{DA}\;\mathrm{in}\;\mathrm{the}\;\mathrm{IL}\;\mathrm{extract}\;\left(\mathrm{mg}\right)}\times 100$$

(3) $$\mathrm{Purity}\;\left(\%\right)=\frac{\mathrm{Total}\;\mathrm{amount}\;\mathrm{of}\;D\;\mathrm{and}\;\mathrm{DA}\;\mathrm{in}\;\mathrm{the}\;\mathrm{product}\;\left(\mathrm{mg}\right)}{\mathrm{Total}\;\mathrm{amount}\;\mathrm{of}\;\mathrm{the}\;\mathrm{product}\;\left(\mathrm{mg}\right)}\times 100$$

#### 3.2.7. Solution-State Nuclear Magnetic Resonance Spectroscopy (Solution-State NMR)

The Solution-state ^1^H-NMR measurements were conducted on 500 MHz and 800 MHz NMR Spectrometer (Advance, Bruker, Billerica, MA, USA) to confirm the chemical structures of D and DA solids precipitated from (BMIm)BF_4_. All Samples were dissolved in CDCl_3_ prior to analysis.

## 4. Conclusions

The present work developed a rapid and efficient method for the separation of D and DA from the roots of *A. gigas* using ILs as extraction solvent. The extraction conditions were optimized by RSM. Compared to other ILs and commonly applied organic solvents such as EtOH, (BMIm)BF_4_ showed a greater extraction ability of 43.32 mg/g (97.06%) for D and 17.87 mg/g (97.12%) for DA. In addition, a mixture of sole D and DA (total purity of 97%) could be obtained very rapidly from *A. gigas*–(BMIm)BF_4_ extraction solution when DW was used as an anti-solvent. The rates of recovery of D and DA were greater than 95% when the ratios of *A. gigas*–(BMIm)BF_4_ extraction solution to DW were greater than 1:5. In conclusion, the developed approach revealed a remarkable effectiveness compared to conventional methods as, great extraction yields, high purity, and satisfactory recovery of D and DA could be achieved simply and rapidly. Through the findings it was confirmed the capability of ILs for the separation of natural products. To our knowledge, this is the first time that an efficient method using ILs in combination with crystallization was achieved for the obtention of high-purity D and DA from *A. gigas*. The developed technique is assumed crucial in the pharmaceutical industry for the effective obtention of high-yield and high-purity D and DA from *A. gigas.*

## Figures and Tables

**Table 1:** Extraction efficiencies of D and DA: design arrangements and responses.

	Parameters			Responses			
Run	A (min)	B (°C)	C (g/mL)	Predicted Y_1_	Observed Y_1_	Predicted Y_2_	Observed Y_2_
1	30 (−1)	40 (0)	4 (−1)	55.67	54.47	55.73	54.69
2	75 (0)	60 (1)	8 (1)	88.41	88.77	90.45	91.37
3	75 (0)	60 (1)	4 (−1)	62.68	62.94	65.12	65.87
4	120 (1)	60 (1)	6 (0)	95.93	94.36	97.74	95.78
5	75 (0)	40 (0)	6 (0)	88.78	87.92	90.65	90.84
6	120 (1)	40 (0)	4 (−1)	77.24	78.54	80.14	81.35
7	75 (0)	20 (-)	4 (−1)	61.21	60.85	62.10	61.18
8	120 (1)	20 (-)	6 (0)	89.31	88.37	92.11	91.82
9	75 (0)	40 (0)	6 (0)	88.78	89.37	90.65	89.91
10	30 (−1)	40 (0)	8 (1)	83.01	81.71	84.84	83.63
11	75 (0)	20 (-)	8 (1)	80.08	79.82	80.85	80.10
12	75 (0)	40 (0)	6 (0)	88.78	89.04	90.65	91.20
13	30 (−1)	60 (1)	6 (0)	77.68	78.62	81.06	81.35
14	120 (1)	40 (0)	8 (1)	94.50	95.70	95.13	96.17
15	30 (−1)	20 (-)	6 (0)	55.67	76.08	74.08	76.04

Where A: Extraction time; B: Extraction temperature, C: Solid-to-liquid ratio, Y_1_: Extraction efficiency of D; Y_2_: Extraction efficiency of DA.

**Table 2:** Variance analysis for the quadratic response surface regression model.

**D Extraction Efficiency**	**Degrees of Freedom**	**Sum of Squares**	**Mean Square**	***F*-Value**	***p*-Value**
Model	9	2146.41	238.49	82.02	0.001
A	1	545.99	545.99	187.78	0.001
B	1	47.87	47.87	16.46	0.001
C	1	994.58	994.58	342.06	0.001
A^2^	1	5.07	0.008	0.00	0.961
B^2^	1	48.18	73.597	25.31	0.004
C^2^	1	464.58	464.58	159.78	0.001
AB	1	2.98	2.98	1.02	0.358
AC	1	25.40	25.40	8.74	0.032
BC	1	11.76	11.77	4.05	0.1
Residual	5	14.54	2.91	-	-
Lack of fit	3	13.38	4.461	7.72	0.117
Pure error	2	1.16	0.58	-	-
Corrected Total	14	2160.95	-	-	-
**DA Extraction Efficiency**	**Degrees of Freedom**	**Sum of Squares**	**Mean Square**	***F*-Value**	***p*-Value**
Model	9	2265.63	251.74	75.65	0.001
A	1	602.22	602.22	180.98	0.001
B	1	79.57	79.57	23.91	0.001
C	1	971.96	971.96	292.10	0.001
A^2^	1	0.00	0.05	0.00	0.961
B^2^	1	70.39	70.391	21.15	0.004
C^2^	1	501.45	501.45	150.70	0.001
AB	1	0.46	0.46	0.14	0.358
AC	1	49.84	49.84	14.98	0.032
BC	1	10.82	10.82	3.25	0.1
Residual	5	16.64	3.33	-	-
Lack of fit	3	15.75	5.25	11.85	0.117
Pure error	2	0.89	0.44	-	-
Corrected Total	14	2282.27	-	-	-

**Table 3:** Verification of the optimized condition: reproducibility and precision.

Experiments	D Yield (mg/g)	DA Yield (mg/g)	D Extraction Efficiency (%)	DA Extraction Efficiency (%)
1	43.18	17.71	96.75	96.27
2	43.36	17.89	97.16	97.23
3	43.42	17.99	97.28	100.7
Average	43.32	17.86	97.09	97.07
RSD (%)	0.2	0.14	0.28	2.35

**Table 4:** Comparison of the (BMIm)BF_4_ extraction method with conventional extraction methods.

Extraction Method	Time (h)	Temperature (°C)	D Yield (mg/g)	DA Yield (mg/g)
(BMIm)BF_4_	2	60	43.32	17.86
60% EtOH Reflux	6	95	29.80	13.55
50% EtOH	5	60	35.46	15.22
100% EtOH	2	60	18.24	8.75

**Table 5:** Supersaturation levels of D and DA in various *A. gigas*–IL extraction solution/anti-solvents (1:5 (*v*/*v*)).

(BMIm)BF_4_/Anti-Solvent (*v*/*v*)	D. C_I_ (mg/mL)	D. C_eq_ (mg/mL)	S_D_	DA. C_I_ (mg/mL)	DA. C_eq_ (mg/mL)	S_DA_
(BMIm)BF4/MeOH	1.11	0.90	1.23	0.42	0.36	1.17
(BMIm)BF_4_/EtOH	1.11	0.95	1.17	0.42	0.33	1.27
(BMIm)BF_4_/DW	1.11	0.21	5.28	0.42.	0.07	6.00

**Table 6:** Supersaturation levels of D and DA in *A. gigas*–(BMIm)BF4 extraction solution/DW (*v*/*v*).

(BMIm)BF_4_/DW (*v*/*v*)	D C_I_ (mg/mL)	D C_eq_ (mg/mL)	S_D_	DA C_I_ (mg/mL)	DA C_eq_ (mg/mL)	S_DA_
1:1	3.11	3.09	1.01	1.32	1.23	1.07
1:3	1.56	0.88	1.77	0.66	0.36	1.83
1:5	1.04	0.21	4.95	0.44	0.08	5.50
1:10	0.57	0.06	9.5	0.24	0.01	24.00
1:15	0.39	0.01	39.00	0.16	0.00	45.52
1:20	0.29	0.01	29.00	0.13	0.00	56.88

**Table 7:** Concentration difference (ΔC) and yields of D and DA depending on the DW composition ratio.

(BMIm)BF_4_/DW (*v*/*v*)	ΔC_D_ (mg)	D Yield (%)	ΔC_DA_ (mg)	DA Yield (%)
1:3	2.70	43.41	1.19	45.25
1:5	4.96	79.74	2.15	81.75
1:10	5.62	90.35	2.52	95.82
1:15	6.06	97.42	2.57	97.71
1:20	6.01	96.62	2.58	98.09

**Table 8:** Total Purity of D and DA in the products.

(BMIm)BF_4_/DW (*v*/*v*)	Total Weight of Product (mg)	Total Content of D and DA (mg)	Total Purity of D and DA (%)
1:5	7.35	7.11	96.73
1:10	8.39	8.14	97.02
1:15	8.85	8.63	97.51
1:20	8.83	8.59	97.28

**Table 9:** Linear ranges, correlation coefficients, LOD, and LOQ of calibration curves.

Analytes	Linearities	Correlation Coefficient	Calibration Range (μg/mL)	LOD (μg/mL)	LOQ (μg/mL)
D	Y = 28.994x − 16.247	0.996	2.5~100	0.316	0.958
DA	Y = 30.314x + 29.732	0.999	2.5~100	0.764	2.316
